# Characterization of Pore Structure in Biologically Functional Poly(2-Hydroxyethyl Methacrylate) - Poly(Ethylene Glycol) Diacrylate (PHEMA-PEGDA)

**DOI:** 10.1371/journal.pone.0096709

**Published:** 2014-05-09

**Authors:** Amelia Zellander, Chenlin Zhao, Mrignayani Kotecha, Richard Gemeinhart, Melissa Wardlow, Jeremiah Abiade, Michael Cho

**Affiliations:** 1 Department of Bioengineering, University of Illinois, Chicago, Illinois, United States of America; 2 Department of Mechanical and Industrial Engineering, University of Illinois, Chicago, Illinois, United States of America; 3 Department of Biopharmaceutical Sciences, University of Illinois, Chicago, Illinois, United States of America; University of California, San Diego, United States of America

## Abstract

A copolymer composed of poly(2-hydroxyethyl methacrylate) (PHEMA) and poly(ethylene glycol) diacrylate (PEGDA) (PHEMA-PEGDA) is structurally versatile. Its structure can be adjusted using the following porogens: water, sucrose, and benzyl alcohol. Using phase separation technique, a variety of surface architectures and pore morphologies were developed by adjusting porogen volume and type. The water and sucrose porogens were effective in creating porous and cytocompatible PHEMA-PEGDA scaffolds. When coated with collagen, the PHEMA-PEGDA scaffolds accommodated cell migration. The PHEMA-PEGDA scaffolds are easy to produce, non-toxic, and mechanically stable enough to resist fracture during routine handling. The PHEMA-PEGDA structures presented in this study may expedite the current research effort to engineer tissue scaffolds that provide both structural stability and biological activity.

## Introduction

While the cell's response to topography has been extensively studied [1], [2], [3], [4], [5], the mechanisms that regulate cell interaction with topography remain to be clearly understood [6]. Manipulation of the cell-topography interaction can potentially be exploited for tissue engineering, including cell alignment and tissue development. Reynolds et. al. demonstrated that cell alignment along the axis of micro- and nano-features is cell type dependent [4]. Multiple cell types displayed alignment in response to linear surface features of different widths and depths. For example, human fibroblasts displayed high levels of cell alignment at a wider range of feature dimensions [4]. More importantly, topographies can alter biological functionality. Myoblasts attached to micropegs showed increased RhoA GTPase and myosin heavy chain II (MYH2) expressions [3] that likely modified cell adhesion. Surface roughness also appears to regulate phenotype-specific protein production in MC3T3 osteoblasts [5]. While previous studies of the cell-topography interaction further advanced our understanding, the substrate preparation was largely labor intensive and generally required specialized equipment for casting or lithography [1], [2], [3], [4], [7]. To increase the efficiency of substrate production, an alternative fabrication process may be warranted. The current study examined a fabrication process that utilizes common laboratory equipment and commercially available products to add topographical features to biomaterials. This particular process, which reduces substrate fabrication time and manufacturing complexity, may also accelerate efforts to better understand and manipulate the cell-topography interaction.

Compared to previously reported poly(2-hydroxyethyl methacrylate)-poly(ethylene glycol) (PHEMA-PEG) structures [8], [9], the combination of HEMA and PEGDA could lead to a wide variety of surface features. Since PEGDA is more hydrophilic than PHEMA [8], small volumes of PEGDA may be used to preserve phase separation in the copolymer and crosslink HEMA. Greater hydrophilicity introduced by PEGDA can suppress phase separation by increasing the solubility of the copolymer chains [8], but the selected ratios of HEMA and PEGDA units are expected to yield a mechanically stable copolymer that is structurally sensitive to solvent or porogen adjustments. Furthermore, varying porogen type and volume is likely to induce noticeable changes in the surface feature size and distribution. The PHEMA-PEGDA structures presented in this study are found to provide a rigorous, inexpensive and reproducible platform to engineer a biocompatible hybrid scaffold and elucidate the mechanisms involved in the cell's response to a substrate's structural features.

## Materials and Methods

### 2.1 Formulation of Porous PHEMA-PEGDA

PHEMA-PEGDA copolymers were created for surface and structural assessment, mechanical characterization, and biofunctional testing. The PHEMA-PEGDA structures were made with 7% w/v PEGDA MW 3400 (Glycosan GS710), 2% v/v PEGDA MW 700 (Sigma-Aldrich, 455008), and 38% v/v HEMA (Sigma-Aldrich 128635). Ammonium persulfate (APS) and N,N,N′,N′-tetramethylethylenediamine (TEMED) initiated the polymerization. Samples were made using following porogens: deionized water (W), sucrose (S), or benzyl alcohol (BOH). Details of the three specific formulations are provided in Table 1.

**Table 1:** Porogens used to generate pores in the PHEMA-PEGDA include water (W), sucrose solution (S), and benzyl alcohol (BOH).

Sample	Deionized Water (diH_2_O)	Sucrose (150% w/v in diH_2_0)	Benzyl Alcohol
**PHEMA-PEGDA (W)**	52%	—	—
**PHEMA-PEGDA (S)**	34%	18%	—
**PHEMA-PEGDA (BOH)**	34%	—	18%

This table shows the % v/v quantity of components used to create the PHEMA-PEGDA structures. The deionized water content (% v/v) listed shows the total aqueous content of the final pre-polymerized mix of monomers and other agents. As porogen types and volumes were adjusted, the quantities of PHEMA and PEGDA monomers remained constant.

### 2.2 Incorporation of Collagen to PHEMA-PEGDA

The PHEMA-PEGDA structures were coated with cross-linked rat tail collagen type I (2 mg/ml) (BD Bioscience). Collagen molecules were crosslinked using N(3-Dimethylaminopropyl)–N′–ethyl-carbodiimide (EDC) (5 mM) and N-hydroxysuccinimide (NHS) (5 mM). Dehydrated scaffolds were incubated in the collagen solution for 2 hrs on ice. The collagen was then allowed to gel at 37°C for 1 hr.

### 2.3 Cell Culture

In preparation for biological testing, copolymeric scaffolds were cut into disks of approximately 7 mm diameter and 0.5 mm thickness. The PHEMA-PEGDA samples were subjected to extensive washing. Scaffolds made with water and sucrose porogens were rinsed in deionized water and ethanol solutions for 6 days, while the benzyl alcohol samples were rinsed in the same solutions for 9 days. Water was used for rinsing because it can effectively remove unreacted agents from PHEMA-PEG scaffolds [9], [10]. Ethanol was also used, since our histology images (not shown) indicated that a more effective rinsing agent was needed to remove sucrose and benzyl alcohol porogens from void spaces in the polymer structure. Since the ethanol swells the gels more than water alone, it may be efficient removing unreacted agents and debris. With detection of faint scent of benzyl alcohol at the end of 6 days rinsing, the scaffolds made using benzyl alcohol porogen were rinsed for additional 3 days. At the end of the rinsing process, samples were dried in an oven at 37°C. Each scaffold was UV sterilized prior to biological testing. Primary human corneal fibroblasts (HCFs) were used for biological testing. Cells were fed every 2 to 3 days with GIBCO MEM alpha (Invitrogen, 12561), supplemented with 10% fetal bovine serum (FBS) (Atlanta Biologicals) and 1% antimicrobial-antimycotic solution (Sigma-Aldrich, A5955).

### 2.4 Cell Quantification and Viability

A fluorescent DNA quantification kit (BioRad) was used to assess cell growth in the PHEMA-PEGDA scaffolds (see Table 1). DNA was extracted from cells using TRIzol per the manufacture's protocol. The PHEMA-PEGDA structures created using three different porogens – water (W), sucrose (S), and benzyl alcohol (BOH) – were tested at week 2. Hoechst 33258 probe was used to quantify the amount of DNA extracted from the copolymer structures. The quantification assay solution, 80 µL, was added to a well of a 384 well plate. Six duplicate wells were used to measure DNA extracted from the PHEMA-PEGDA structures. Fluorescent signals were detected and quantified using a spectrophotometer. Acellular scaffolds coated with collagen type I were placed on top of a layer of confluent primary human corneal fibroblasts HCFs that were grown to confluence in petri dish. This approach allowed maximizing active cell attachment, migration and growth.

An alamarBlue assay (Invitrogen) was used to evaluate HCF proliferation in the water porogen PHEMA-PEGDA scaffolds with increasing water content. The structure was made using 59% total deionized water, an increase in water content compared to the PHEMA-PEGDA (W) listed in Table 1, to determine how increasing the porogen volume affects the pore size. Initially, 6×103 HCFs were added to the surface of the collagen type I coated structure. The surface area of each circular polymer disk was approximately 160 mm2. Confocal microscopy was used to view live HCFs on the surface of collagen type I coated scaffolds. Prior to imaging, cells were loaded with a cell viability indicator (Invitrogen L3224).

### 2.5 Tension Testing

**Figure 1:**
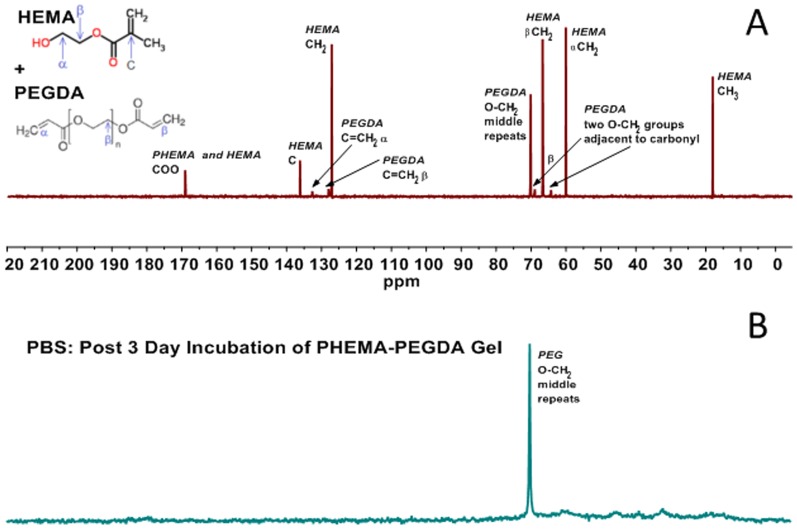
NMR spectroscopy results indicate that PEG chains appear to have leached out of the PHEMA-PEGDA scaffold following 3 days soaking in PBS. The signal for the middle O-CH2 group of PEGDA (A) and the middle O-CH2 group of PEG (B) were both found at approximately 70 ppm. Signals that represent the repeating side groups in PHEMA were not observed in PBS, however, indicating neither HEMA nor PHEMA leached out.

The PHEMA-PEGDA structures were tested in tension using a custom designed mechanical testing apparatus. Three samples of each formulation were tested using a calibrated WF75GS Load Cell (Test Resources Inc.), which is fatigue-rated for 75 g of force (0.735 N) in tension and compression. The polymer structures were made in dumbbell shaped molds for tension testing according to the ASTM specification (D638 Figure 1, Type IV). First, a strain of 2–5% was applied for 30 cycles to pre-condition the samples. Next, elastic modulus was calculated using a 2–10% strain for 30 cycles. Strain rate was approximately 1 mm/s for all elastic modulus measures. Mid-range cycles, typically 10–20, were used to compute the elastic modulus. Tension was then applied to other dumbbell shaped polymer samples until mechanical rupture was achieved. The scan rate for the force measure was 50.86 Hz. Damping factors were calculated using phase shifts between the stress over time and strain over time curves. The tangent of the horizontal phase shift corresponds to the energy loss of the copolymer per tension cycle; the value is known as the damping or dissipation factor] [11]. Phase shifts were measured using MATLAB.

### 2.6 Structural and Chemical Characterization

Scanning Electron Microscopy (SEM) images were generated using a S-3000N Variable Pressure SEM (Hitachi). Low magnification SEM images were taken of uncoated PHEMA-PEGDA. Prior to high magnification imaging, structures were laser ablated with gold using a Pulsed Laser Deposition System (Excel Instruments). Images were used to evaluate the effects of porogens on the surface textures and structures. Surface pore areas and shapes were digitally processed and thresholded in preparation for measurement. Each pore area was measured with the ImageJ 1.47c.

Nuclear magnetic resonance (NMR) was used to evaluate the stability of the PHEMA-PEGDA scaffold in aqueous solution. A PHEMA-PEGDA (water porogen) gel was incubated in PBS for 3 days. Then, NMR measures were taken from both the PBS soaked gel and a solution of HEMA and PEGDA monomers. The ratio of HEMA to PEGDA in the monomer mixture was equal to the ratio of monomers used to create PHEMA-PEGDA gels. All 13C NMR measurements were acquired using the “zgpg” pulse program at room temperature of a 8.5 T (1H frequency  = 360.13 MHz and 13C frequency, 90.55 MHz) Bruker Advance spectrometer equipped with a QNP probe capable for multinuclear NMR measurements. A capillary filled with D2O was immersed in NMR tube for locking the deuterium signal. The solid gel samples were immersed in PBS for three days and placed on top of a doty aurum plug (14 mm) to keep the samples at the center of RF coil. The 90° pulse width was 13.5 µs for monomer solutions and was used as it is for polymer samples. The relaxation delay in all experiments was set to 2 s. The spectra were acquired with 1024 scans (signal accumulations) as compared to only 4 scans for liquid spectra.

### 2.7 Statistical Analysis

MATLAB R2012a was used to perform all statistical analysis at an alpha level of 0.05. The statistical significance of mechanical data was evaluated using ANOVA. Kruskal-Wallis was used to evaluate the statistical significance of DNA concentrations. Data from alamarBlue was evaluated using a two sample t-test. All reported values represent mean ± SE.

## Results

### 3.1 Chemical Assessment of PHEMA-PEGDA

High resolution 13C NMR spectra at 8.45 T (1H freq. 360 MHz) were obtained for a solution of unpolymerized HEMA and PEGDA and a PHEMA-PEGDA hydrogel soaked in PBS for 3 days. For correct peak assignments, 13C NMR spectra of HEMA and PEGDA solution were also acquired individually (data not shown). The peak assignments for spectral lines are based on both published literature [12], [13] and general NMR references [14], [15].

The HEMA and PEGDA units in the solution are not chemically connected as all of the peaks can be assigned to individual components (Fig. 1A). In contrast, the spectra of PHEMA-PEGDA hydrogel following a 3-day incubation in PBS showed all the individual peaks disappeared with exception of one peak (Fig. 1B). Since the nuclei in gel polymers have stronger dipolar couplings with other nuclei, one would not expect to see any signal from the gel soaked in PBS if the monomer units were all polymerized. However, the middle repeat O-CH2 group of PEGDA was found to be present at 70 ppm in the spectra with the line width of approximately 20 Hz (the line width of 6 Hz observed in liquid spectra), which indicates that PEG units may have leached out of the PHEMA-PEGDA hydrogel. Another possibility is that this middle unit of PEGDA is flexible and mobile. Neither HEMA nor PHEMA appeared to have leached out, as none of the peaks from HEMA are present in the PBS soaked gel spectra.

### 3.2 Testing Porous PHEMA-PEGDA in Tension

**Figure 2:**
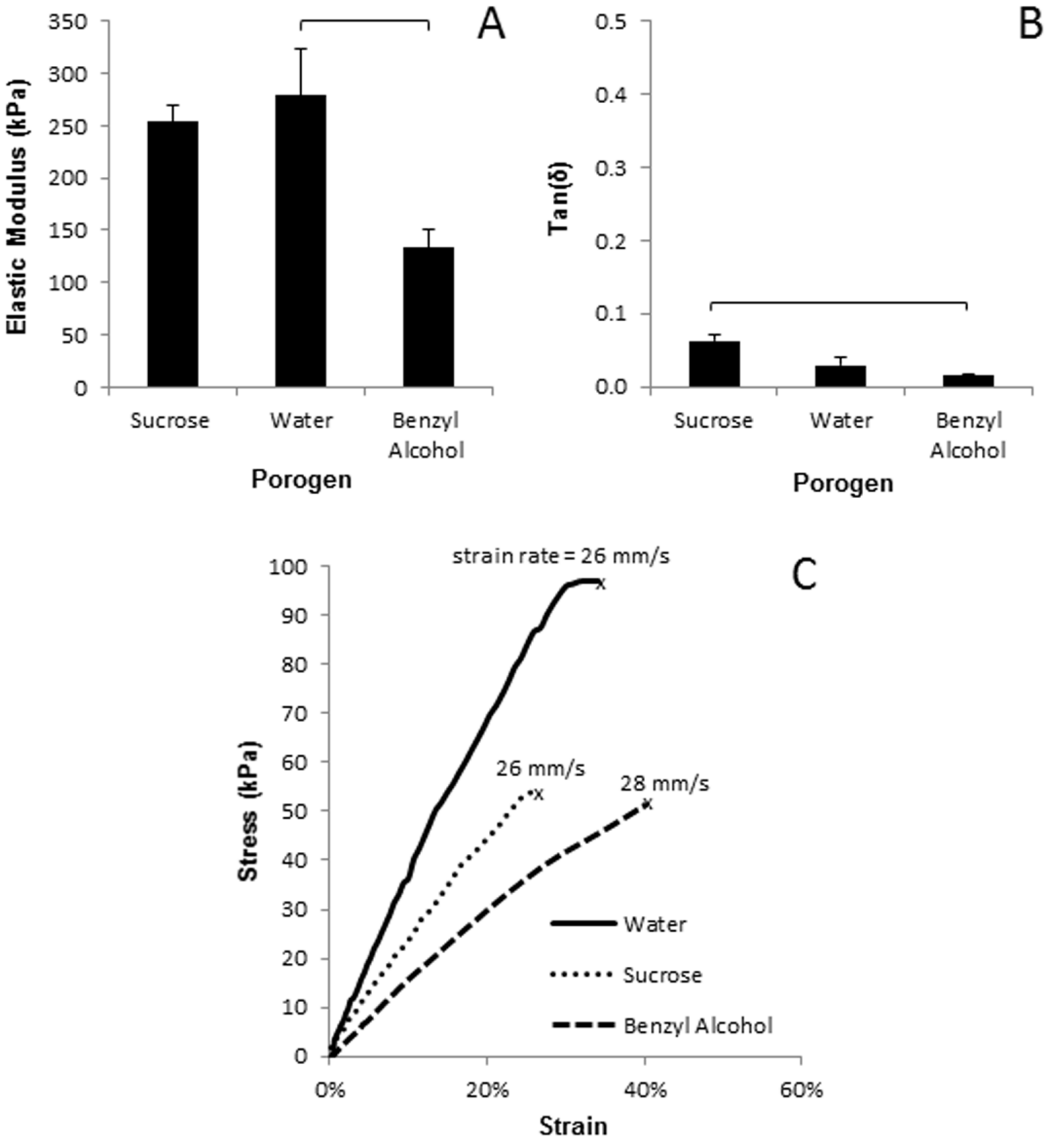
Tensile testing showed that the porogens can significantly alter the mechanical properties. The PHEMA-PEGDA made with water porogen was significantly stiffer than samples made with benzyl alcohol porogen. Low damping factors (tan (δ)) show that the PHEMA-PEGDA samples are viscoelastic with dominant elastic properties (B; n = 3). Tension was applied until rupture to determine the ductility and overall strength of the scaffolds (C). “X” marks the failure or rupture point of each sample. Horizontal bars identify samples with a statistically significant difference (p<0.05).

The porogen type influenced the mechanical properties of PHEMA-PEGDA. The PHEMA-PEGDA made with either water or sucrose porogen (designated W and S, respectively) was significantly stiffer (∼275 kPa) than the PHEMA-PEGDA made with benzyl alcohol porogen (BOH, ∼140 kPa; Fig. 2A). Damping factors (i.e., tan(δ)), measured at room temperature and at 1 mm/s strain rate, show that the porogens had a statistically significant effect on viscoelasticity. Note that damping factor, which is a function of temperature and frequency of strain application [11], for the sample made with sucrose porogen was significantly higher than that made with BOH. However, all three samples were found viscoelastic with dominant elastic properties (low energy loss; Fig. 2B). A perfectly elastic material would have a damping factor of 0 [11], [16]. The largely elastic PHEMA-PEGDA hydrogels are expected to maintain their mechanical properties during experimental handing involved in tissue engineering. For example, these samples comfortably resisted rupture at 25 kPa and 20% strain (Fig. 2C).

### 3.3 Surface Texture of PHEMA-PEGDA

**Figure 3:**
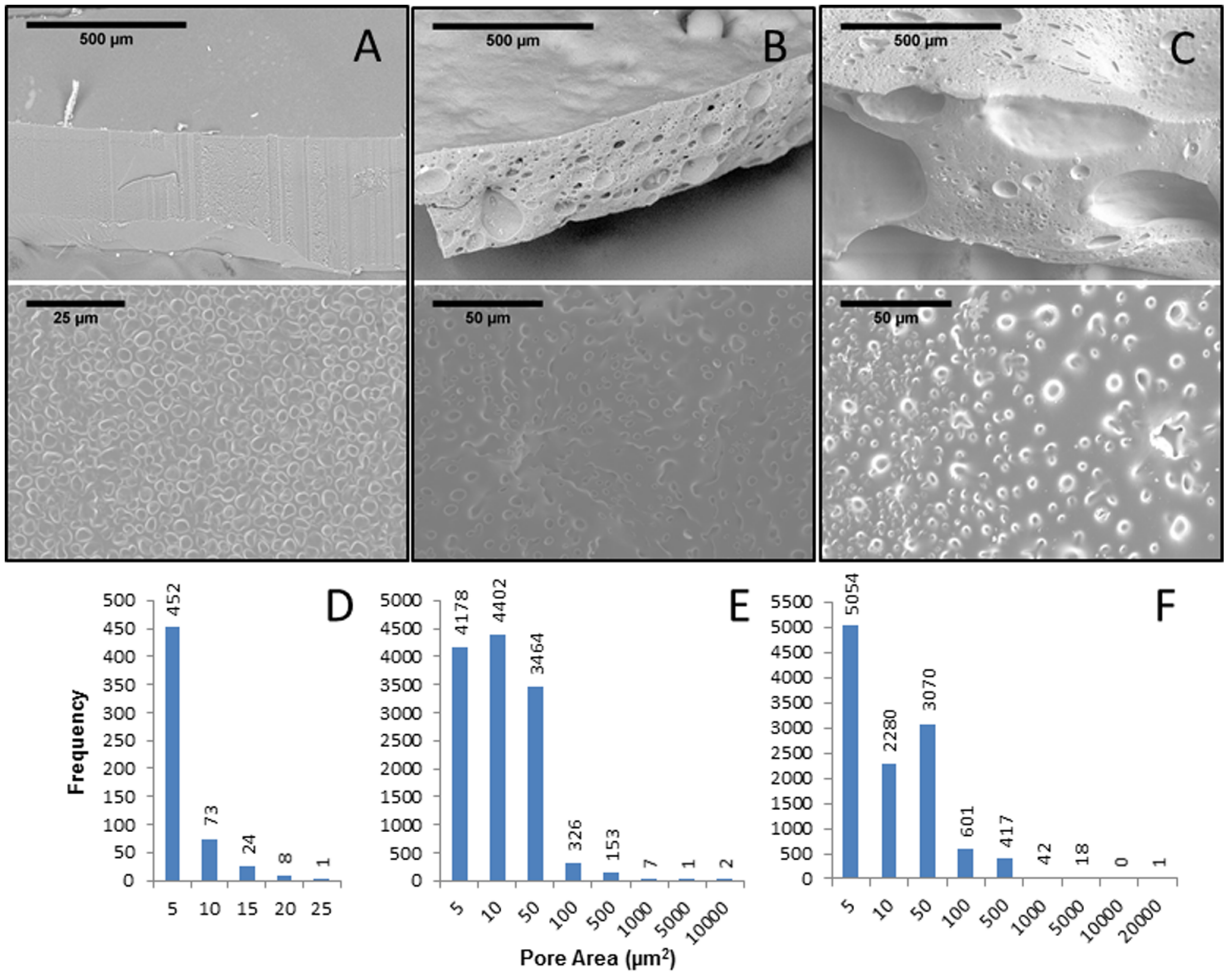
SEM images show dehydrated PHEMA-PEGDA. Water (A), sucrose (B), and benzyl alcohol (C) were used as porogens. Low magnification SEM images are displayed in the top row. Corresponding SEM images with higher magnification are shown in the bottom row. Image analysis was used to measure pore areas. Histograms display pore areas for the scaffolds made with water (D, n = 558), sucrose (E, n = 12,533), or benzyl alcohol (F, n = 11,485) porogens.

SEM images were analyzed for surface pore areas created by the three porogens. Image analysis indicates that the three porogens produced different ranges of pore areas. For example, the pores resulting from the water porogen demonstrated the smallest range of pore areas (1–21 µm2; Fig. 3A and 3D), and more than 90% of pores analyzed had the pore size <5 µm2. Because both the sucrose and benzyl alcohol appear to produce a variety of pores, we increased the number of pores to >11,000 to construct a distribution histogram. The sucrose porogen generated pore areas ranging from 7– µm2 but ∼96% of the pores were <50 µm2 in size (Fig. 3B and 3E). While the benzyl alcohol produced the largest range of pore sizes, the image analysis showed that still >90% of the pores were <50 µm2 in size (Fig. 3C and 3F). Although generating SEM images required dehydration of the samples, we observed that larger pores can be seen upon rehydration. Control over the porogen type and volume can exhibit heterogeneous pore structures.

### 3.4 Cellular Response to Copolymers

**Figure 4:**
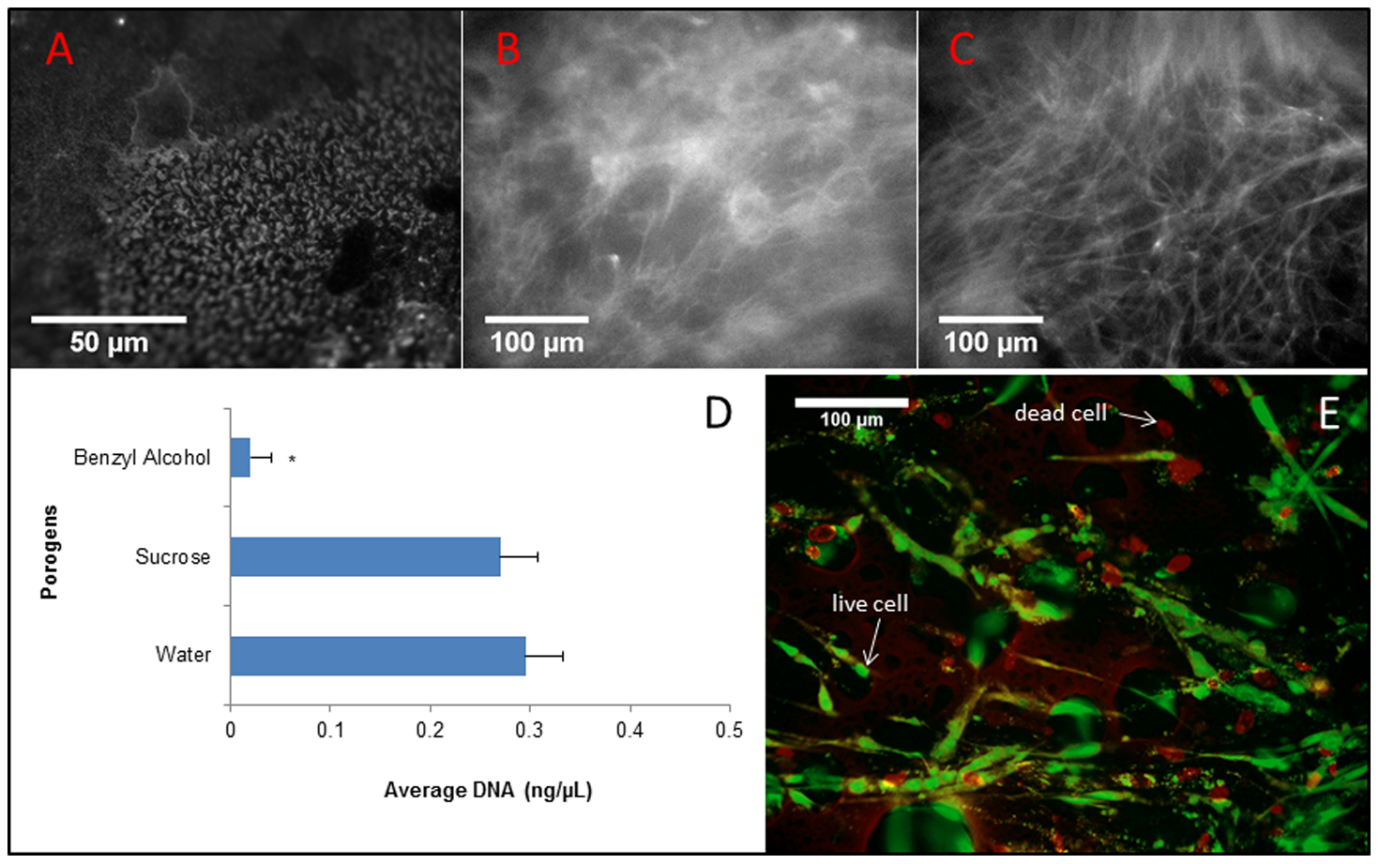
Collagen type I coating the PHEMA-PEGDA scaffolds was immunostained using collagen antibody. Scaffolds were removed from the tissue culture wells and rinsed with PBS. (A) Collagen gelled within the micron sized surface pores of PHEMA-PEGDA using the water porogen. Collagen fibers were evident in the sucrose and benzyl alcohol PHEMA-PEGDA scaffolds (B and C, respectively). At week 2, HCFs were detected in the three different PHEMA-PEDGA scaffold types, and average DNA contents were quantitatively measured (D; n = 6). “*” indicates p<0.05, and error bars represent standard error. Cell viability staining at day 7 shows both live (green) and a significant number of dead (red) HCFs in the benzyl alcohol PHEMA-PEGDA scaffold (E).

For tissue engineering applications, one of the important criteria for scaffolds is their capability to accommodate living cells. Because combinations of PEG and PHEMA may potentially be used in artificial corneas [17], we used human corneal fibroblasts (HCFs) to evaluate cytocompatibility. The PHEMA-PEGDA hydrogel was coated with collagen type I to promote cell adhesion to the surface features. Collagen antibody staining confirms the presence of collagen fibers in these scaffolds (Fig. 4A–C). It appears that the collagen fibers were incorporated within the surface pores of PHEMA-PEGDA. Since cell-free scaffolds were initially placed on the top of HCFs, the DNA quantification data showed that cells migrated into the collagen coated polymers (Fig. 4D). A comparison of the two porogen types, water and sucrose, demonstrated that the two structures supported essentially equivalent cell attachment. In contrast, a significantly fewer number of cells was attached to the structure made with the benzyl alcohol porogen. Figure 4E shows live and dead HCFs on the surface of scaffolds made with benzyl alcohol porogen, confirming the DNA measurement.

### 3.5 Adjusting Porogen

**Figure 5:**
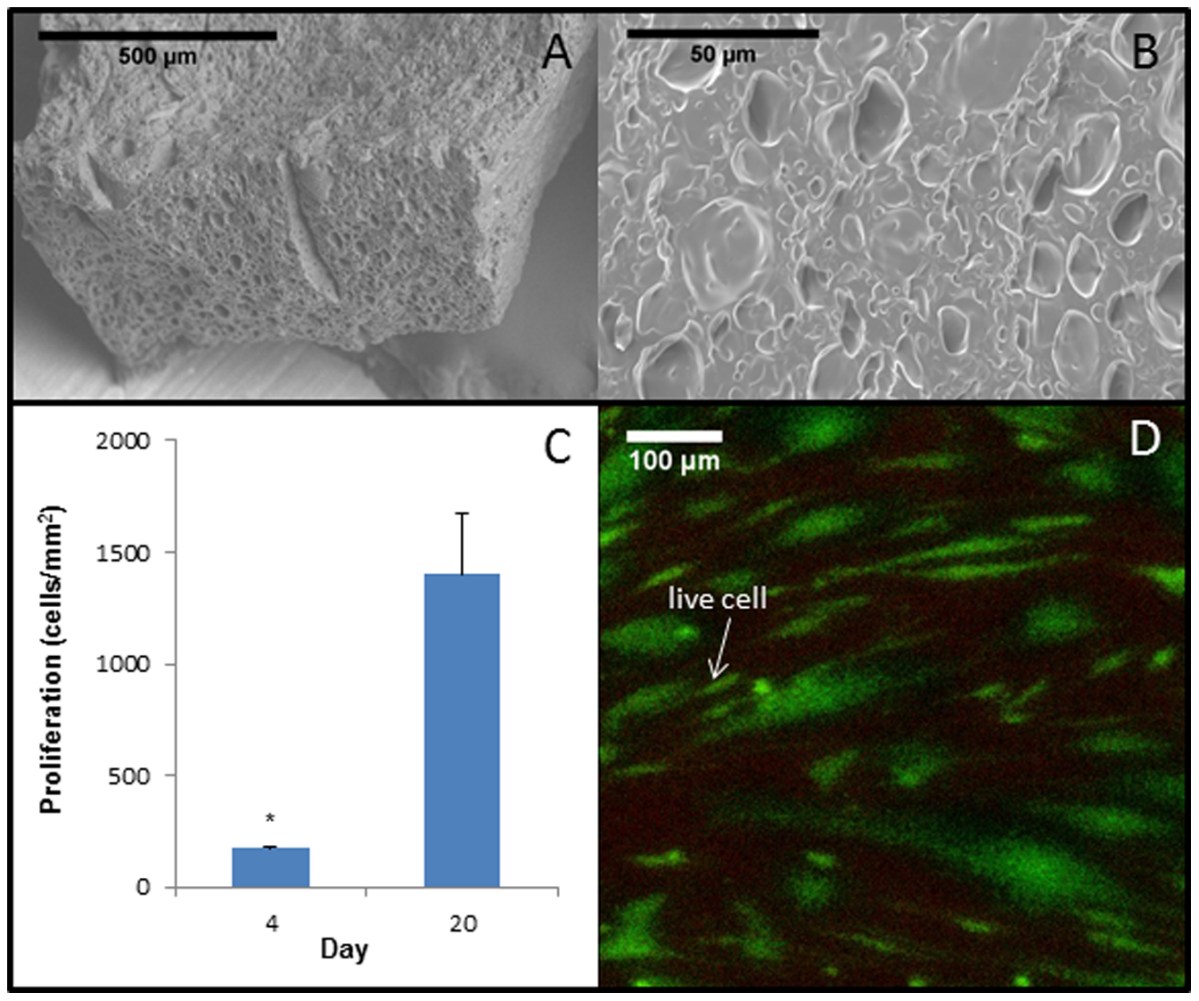
SEM images of PHEMA-PEGDA scaffolds in low (A) and high (B) magnification. By increasing the content of the total deionized water content by 7%, average pore area was substantially increased. Data from alamarBlue shows that HCFs proliferated significantly in this structure from day 4 to 20 (C, p<0.05, n = 3). Fluorescent images show that, at day 8, live HCFs were clearly visible in this scaffold with essentially minimal cell death (D).

We next examined the relationship between the pore size and porogen volume for the most cytocompatible porogen (e.g., water). We observed that small adjustments in the total deionized water content of the PHEMA-PEGDA resulted in notably larger surface pore areas. For example, by increasing the water content from 52% to 59% (see Table 1), this 7% increase in water yielded an average pore size (∼50 µm2, Fig. 5A) that is an order of magnitude larger. Data from alamarBlue shows that HCFs also adhered to this structure. As shown in Figure 5B, a significant increase in the number of cells adhered to this modified PHEMA-PEGDA structure was observed from day 4 to day 20 (n = 3). Live cells on the structure are displayed in Figure 5C. Increasing further the total water content to 65% eliminated surface pores at the micron level and produced wave-like features on the surface of the structure (images not shown). Adjustments of the content of water porogen between 52% and 65% may result in surface pore sizes and patterns that could be useful for the cell-topography investigation.

## Discussion

Different PHEMA-PEGDA formulations presented here offer a range of surface pore sizes compared to previously published reports [8], [9], [10], [18]. Horák et. al. created porous PHEMA using sodium chloride (NaCl) and sucrose. Pores made with NaCl were up to 9 µm in diameter (∼28 µm2), and pores made with sucrose were up to 26 µm in diameter, demonstrating that the PHEMA pore size depends on the type and volume of both crosslinkers and porogens [18]. Dziuble et. al. created PHEMA - poly(ethylene glycol) monomethacrylate (PEGMA) with average pore diameters between 7 to 16 µm [10]. In addition, Baker et. al. created surface pores as large as 50 µm in PHEMA- poly(ethylene glycol) methyl ether methacrylate (MeO-PEGMA) using NaCl [8]. However, our study presents an alternative cytocompatible structure that provides a different range of pore sizes (Figs. 3–5). Water and sucrose porogens can be adjusted to produce surface pores in PHEMA-PEGDA, and the estimated pore diameter may be as large as ∼70 µm when the surface pore areas are assumed to be circular.

Following the sample washing process, the PHEMA-PEGDA made with benzyl alcohol porogen was not expected to adversely affect cells. Benzyl alcohol is a linear aryl alkyl alcohol that is used as an anti-microbial agent at 3–5% [19], [20]. It is neither mutagenic nor carcinogenic, and it occurs in nature in allium plants. However, benzyl alcohol can be harmful in certain quantities. For example, its intravenous LD50 and intraperitoneal LD50 for mice are 0.038 g/kg and 0.65 g/kg, respectively [21]. The structure created with benzyl alcohol porogen resisted HCF migration in comparison to using water or sucrose porogen (Fig. 4D). In addition, more dead cells were observed with benzyl alcohol porogen (Fig. 4E) compared to water porogen (Fig. 5C). Traces of benzyl alcohol residue may have impeded cell migration onto the scaffold. Another potential explanation for the observation could be the effect of substrate stiffness on cell migration and proliferation. The PHEMA-PEGDA (BOH) has the lowest elastic modulus (Fig. 2) and the least number of cells in the scaffold by the 2-week time point (Fig. 4). Ghosh et. al. demonstrated that the migration speed of adult human fibroblasts decreased with increased substrate stiffness, but cell proliferation increased with increased substrate stiffness [22]. While a more effective washing procedure could perhaps remove the benzyl alcohol residues, the two other porogens may be utilized without much concern regarding the effect of residues and still engineer comparable PHEMA-PEGDA scaffolds. Experiments with the porogen types and volumes indicate that further work may reveal additional pore structures that can be created with selected quantities of water and/or sucrose porogens.

The pore diameters in the PHEMA-PEGDA copolymers depend on the solvent fraction. Small pores, approximately 2 µm in diameter (∼3 µm2), were observed in structures made with 52% v/v deionized water in the PHEMA-PEGDA (W) scaffold. At the aqueous solvent content of 59% v/v, the pore sizes increased close to 50 µm2, but then sizes reduced when the aqueous content further rose to 65% v/v. Similar patterns were observed in the PHEMA-PEGDA made with sucrose and benzyl alcohol porogens. Previous studies show that hydrogels made with low aqueous content HEMA [23] or HEMA-PEGMA [8] solutions result in structures with pores that are impenetrable to cells. PHEMA is likely to drive the formation of pores in the PHEMA-PEGDA. Pores in the PHEMA-PEGDA are formed as polymers fall out of the solution during polymerization. The Gibbs free energy of mixing (ΔGM) affects the ability of a polymer to remain in solution and the resulting pore structure of a fully polymerized hydrogel. When ΔGM<0, a homogeneous polymer solution exists. As monomers are converted to polymers in a free radical polymerization, the Gibbs free energy of polymerization (ΔGP) is negative to make polymerization thermodynamically favorable. One can write: ΔGM = G12−(G1–G2), where G12 is the Gibbs free energy of the solution, and G1 and G2 are the Gibbs free energy of solution components. Negative ΔGP is related to the polymer solution and a solution component. Therefore, ΔGP<0 may drive the ΔGM above 0 and cause polymerizing units to fall out of the solution. The pore structures that result from given solvents and additives are also related to ΔGM. ΔGM is then a function of solvent-polymer interaction. The Flory-Huggins interaction parameter (χ) describes the difference in energy between a pure polymer and a polymer immersed in pure solvent. Since ΔGM is a function of χ, the resulting voids that form as polymerizing chains fall out of solution are a function of solvent and polymer interaction [16].

The topographical features of substrates affect both in vitro and in vivo cell activity. Cell membrane alteration caused by surface features may initiate the cell's response to a given topography [6]. Previous studies have identified a relationship between the cell activity and substrate pore size [24], [25]. For example, chondrocytes produced more sulfated glycosaminoglycans (GAGs) on scaffolds with 160,000 µm2 pores compared to those with 10,000 µm2 pores [24]. Vascular cell growth was greater in structures with 38–150 µm diameter pores compared to smaller pores (less than 38 µm in diameter) [25]. Our study validates a non-toxic and mechanically stable copolymer that can be adjusted to create a variety of surface topographies including pores, waves, and bulbous projections. Versatility of the surface features is achieved by varying the porogen types and quantities. As of this date, we are not aware of unique copolymeric HEMA and PEGDA scaffolds that display the level of structural versatility that was presented here. This study expands the current understanding of the liquid porogens that can modify the topography of PHEMA-PEGDA biomaterials.

## Conclusions

Several PHEMA-PEGDA structures with a range of surface features were developed by adjusting porogen types and quantities. These structures provide a variety of surface feature sizes and morphologies of different mechanical properties that could be used to investigate how topography influences cell activity. Surface features include pores, waves, and bulbous projections. Future work may reveal additional surface features that can be created with selected quantities of water and/or sucrose porogens. Structures made using this convenient fabrication process could potentially expand our understanding the mechanisms involved in the cell response to the external mechanical and topographical environment.
